# Supplementary material for: Genomes of Thaumarchaeota from deep sea sediments reveal specific adaptations of three independently evolved lineages
Source: ISME J. 2021 Apr 1;15(9):2792–808. doi: 10.1038/s41396-021-00962-6 (PMC8397731; doi:10.1038/s41396-021-00962-6)
Supplement: Supplementary file 1 — Supplementary Information [file 41396_2021_962_MOESM1_ESM.docx]

**Supplementary information**

**Site description of deep-sea seafloor samples**

All sediment cores used in this study were retrieved from bathyal or abyssal depths. The two Pacific cores are fully oxic, whereas dissolved oxygen was not detectable in the middle part of NP_U1383E and the basal part of GS14-GC08 (Fig. S1). All except one (GC08-250 cm) of the metagenome sequencing datasets were generated from oxic sediment where nitrate was also detected in the porewater (Fig. 1b).

**Amplicon sequencing and analysis**

16S rRNA gene amplicons of the Atlantic sediments were prepared using primers Uni519f/806r and sequenced using an Ion Torrent Personal Genome Machine described previously [1]. The amplicons of the Pacific samples were prepared using a universal primer set of U530F and U907R and sequenced using Illumina MiSeq platform, following the procedure described in [2, 3]. To study the overall community structure of Thaumarchaeota, the sequencing data were processed as described elsewhere [1]. Briefly, the reads were quality-controlled and OTUs (97% nucleotide similarity threshold) were clustered using USEARCH and classified using CREST. For individual cores, OTUs classified as Nitrosopumilales were extracted from the OTU tables, and their clade affiliations were assigned based on their placement in the Nitrosopumilales 16S rRNA gene phylogenetic tree presented in [4].

**High similarity between genomes from the Pacific and Atlantic sediments**

Although the NP-iota MAGs (NPMR_S100_NP_iota_1 and YK1309_1N_S300_NP_iota) were assembled from metagenomic datasets of marine sediments in the Pacific and Atlantic oceans respectively, these MAGs showed 99 % ANI (Fig. S1), suggesting that these bins might represent different strains of the same species (prokaryotic species usually show > 95 % ANI among themselves [5, 6])⁠. A similar pattern was observed between the Pacific bin YK1312_12N_S200_NP_theta and the Atlantic bin NPMR_S100_NP_theta_3, exhibiting > 97 % ANI. It has been shown that prokaryotes with close phylogenetic affiliation inhabiting deep marine sediments and subsurface oceanic crust are commonly retrieved in distant geographic locations [7, 8]⁠, possibly due to the circulating seawater that allows the dispersion of subsurface and benthic microbial phyla [9]⁠.

**Taxonomic placement of NPMR_NP_delta_1**

Based on the *amo*A tree MAG NPMR_NP_delta_1 clustered within the NP-theta clade (Fig. 2b) but the more robust phylogenomic analysis strongly suggests that it belongs to the NP-delta subclade (Fig 2a). We argue that the *amo*A gene in this MAG (in which *amo*AXC are the sole genes of a small contig) might represent a contamination considering that NP-delta and NP-theta have similar ecological distribution [4];⁠ this study) and the phylogenomic tree reconstruction with 79 single-copy gene markers shows clearly that this MAG is placed within the NP-delta lineage.

**Notes on the evolution of AOA lineages from comparative genomics**

Interestingly, our estimation of 269 AOA-specific core families is close to the estimation of 289 protein families inferred to have been gained by the last common ancestor of AOA [10]. We observed that extensive differential gene loss (and possibly gene acquisitions) have occurred at the origin of major AOA lineages as is attested by the great number of families shared between different combinations of protein families present in 2, 3 or 4 lineages only (Fig. 3, S2). Similarly, we found that each AOA lineage harbors between 500 to more than 3000 lineage-specific families suggesting a complex genomic evolution and a considerable number of gene acquisitions at the origin and during the diversification of AOA lineages, possibly associated to habitat adaptations [10–13].

Based on genomic context analyses and considering the phylogenetic position of the NP-iota clade in the context of the scenario proposed by [14], it is plausible that the V-type ATPase was acquired by the common ancestor of NT/NP clades, followed by selective losses of one or the other in the resulting clades according to their environmental radiation. Genomic context analysis reveals that in NP-iota MAGs, the two operons are encoded next to each other, and flanked by the pyrI, pyrB and sulfT genes which are also the flanking genes of the V-type ATPase operon in NT and NP-alpha genomes/MAGs, as well as the A-type ATPase operon in all other NP clades [14]. In abysso/hadopelagic NP-gamma AOA, the V-type ATPase operon is located elsewhere in the genome (Table S2), implying an independent acquisition [14]. In any case, this distribution suggests that the acquisition of the proton-pumping ATPase variant was crucial for successful radiation into hadal high-pressure environments and raises intriguing possibilities about the ecophysiological potential of the ancestor of *Nitrosopumilales* (see evolution scenario above).

**Temperature range inference for the deep marine sediment AOA**

Our presumption regarding the putative temperature growth range (mesophilic/psychrophilic/psychrotolerant) of the deep marine sediment AOA is based on the following lines of evidence:

- The identification of cold-shock proteins (CspC & CshA) in the sediment clades, homologs of which are not found in thermophilic AOA (or thermophilic microorganisms in general), and conversely the absence of thermophilic signature genes such as reverse gyrase or cDPG synthesis genes (the later found in *Ca.* Nitrosocaldales)
- The temperature growth range of cultivated NP members encoding CshA (members of the genera *Nitrosopumilus, Nitrosoarchaeum, Ca.* Nitrosopelagicus)
- AOA represented by these genomes were shown to present throughout core U1383E (spanning over 8 M years) and even show net growth in the oxic-anoxic transition zone of an estimated temperature of 2.05 (1.5 +22*25/1000) [1].
- The amoA gene survey of Alves et al. (2018) also indicate that these AOA (especially the NP-theta clade) are mainly present in marine sediments, with most of the surveyed samples stemming from depths with low *in situ* temperatures [15].
- Studies using metatranscriptomics, isotope labeling, detection of intact archaeal lipids and other techniques have repeatedly shown active Thaumarchaeal populations in marine subseafloor sediments [16, 17] and deep sea samples [18] (all references are in main text)

**Usage of exogenous organic compounds and high pressure adaptations**

The thaumarchaeal putative lactate racemase family enzyme has a 32% amino acid identity (1e-59) to the characterized LarA from *Lactobacillus plantarum*, and is a nickel-dependent enzyme activated by a maturation system [19] also found in the sediment AOA bins (Table S2, S4). In lactobacilli, D-lactate is an important cell wall component conferring resistance to vancomycin [20], a glycopeptide antibiotic which inhibits cross-linking of N-acetylmuramic acid (NAM)/ N-acetylglucosamine (NAG) polymers. NAG/NAM is also a component of the thaumarchaeal cell surface, given the presence of NAG-utilizing enzymes in AOA (Tables S2, S4, [21]). The presence of Lar would enable the utilization of both lactate stereoisomers produced by the sediment fermentative community. The transport of lactate could be mediated by MIP family transporters (aquaporins) [22] common in AOA, as in lactobacilli. It has to be noted though that this enzyme belongs to a large superfamily of proteins with broad distribution in non-lactate utilizing organisms, probably catalyzing other racemization reactions [20, 23]. Phylogenetic analysis of the superfamily PF09861 of which LarA is a member reveals that the AOA homologs belong to a separate, but neighboring, cluster from the characterized LarA homologs from lactobacilli, leaving the question of the putative substrate open (Fig. S6)

The malate dehydrogenase (MDH) homologs encountered in AOA belong to the LDH-like MDH subgroup within the LDH/MDH superfamily of 2-ketoacid:NAD(P)-dependent dehydrogenases, as other archaeal MDHs [24]. In terms of primary sequence, quaternary structure and enzymatic properties, characterized archaeal homologs are between canonical MDHs and LDHs, possessing clear activity with oxaloacetate (as the former) but also able to utilize pyruvate (as the latter), while also exhibiting relaxed cofactor specificity (NADH or NADPH) [24, 25]. The active site architecture surrounding the universally conserved substrate binding residue (Arg171) resembles the environment found in canonical LDHs in AOA homologs, as in the characterized LDH-like MDH from *Ignicoccus islandicus* (Fig S5) [24]. In particular, while position 102 is occupied by an arginine and a neutral residue (methionine) is found in position 199, as in canonical MDHs, a threonine at position 246 and a histidine at position 68, typical LDH residues, may influence substrate selection and charge balance respectively (Fig. S5, residues highlighted in orange) [24, 25]. We therefore hypothesize that AOA homologs exhibit a broad substrate specificity and are in principle able to convert lactate to pyruvate, with the concomitant formation of NADH (cofactor preference for NADH is inferred by the presence of Asp54, highlighted in green in Fig. S4). However, whether this actually takes place *in vivo* necessitates enzymatic characterization.

No genes associated with the glycine cleavage system or choline/betaine degradation present in certain hadopelagic NP-gamma and NP-alpha lineages [26, 27] were identified in any of the sediment bins (Fig. S4).

Interestingly, a part of the NADH dehydrogenase (complex I) operon is duplicated in two out of three NP-delta MAGs (Fig. 5, Tables S2, S4) specifically genes nuoIJKML in NPMR_NP_delta_1 and nuoHIJKM in NPMR_NP_delta_3, bearing 85-95% amino acid identity. Unfortunately, these duplicated regions are in single contigs and therefore it is unclear whether the whole operon is duplicated. It is intriguing, however, that these regions contain the proton pumping subunits of complex I, raising the possibility that this could either be a mechanism to alleviate cytoplasm acidification under high pressure if the complex is running in the forward direction and pumping protons out, similar to the V-type ATPase. It should be noted here that Complex I is postulated to run in reverse in nitrifiers [28, 29]. Duplicated subunits or multiple copies of complex I are observed in various microorganisms including members of AOB and NOB, and are associated with increasing proton-pumping capacity or providing different electron flow options by operating in different directions, respectively [30–32]. Facultative piezophiles such as *Shewanella violacea* has been shown to encode distinct complexes of the respiratory chain (such as different versions of terminal oxidases) as an adaptation to growth at different pressure conditions [33].

**Usage of amino acids (AA)**

Enzymes participating in canonical amino acid biosynthesis pathways and present in almost all AOA (e.g. aspA, ilvA, ilvE, aspC glyA, GDH) could enable the utilization of imported amino acids such as Asp, Gly, Ser, Thr, Ile, Val, Leu, Phe, Tyr into their corresponding α-ketoacids or dicarboxylic acids by releasing a molecule of ammonia (Fig. 5, S4, Tables S2, S4). Only the catabolism of proline to glutamate by proline dehydrogenase (ProDH) and 1-pyrroline-5-carboxylate dehydrogenase (RocA), two enzymes with conserved gene synteny, is not widespread in AOA but present only in certain clades (Fig. 5, S4). Proline catabolism would generate reducing equivalents (Fig. 5) and result in the production of glutamate, which can be used to regulate the ammonia pool via the action of glutamate dehydrogenase (GDH) or would have a possible role in osmoregulation [34]. A type-III aminotransferase (Oat, CLUSTER_3296) encoded by the NP-theta, NP-iota MAGs could also participate in replenishing the intracellular ammonia stock by catalyzing the transamination between a variety of amino acids, mono- and diamines and α-ketoacids (Fig. 4, 5).

In obligate piezophiles such as *Thermococcus barophilus*, a drastic increase in AA requirements during HHP growth, even for those where biosynthesis pathways are present, was interpreted as a switch from energy-intensive AA biosynthesis towards recycling, in the context of the general metabolic response towards more efficient energy utilization under HHP [35–38]. A downregulation of AA biosynthesis pathways (especially glutamine, glutamate and costly aromatic AA), together with an upregulation of AA transport systems is observed during HHP growth in transcriptomic studies of facultative piezophiles *Desulfovibrio hydrothermalis* and *Desulfovibrio piezophilus*, While this was interpreted as solely resulting in glutamate accumulation in the cells (acting as a piezolyte), the accompanying shift in the energy metabolism of these organisms towards increased energy efficiency could also be taken as evidence of AA recycling. Moreover, in obligate piezophiles such as *Pyrococcus yayanosii*, certain AA biosynthesis pathways have been altogether lost [39], while *P. abyssi* also requires 9 amino acids for growth despite possessing biosynthesis pathways [40].

**Transporter complement of sediment clades**

All deep sediment clades encode the ion transporter repertoire typical for marine microorganisms (Tables S2, S4, Fig S4)([26–28, 41–44]: aquaporins (MIP family), small conductance mechanosensitive channels (MscS, accompanied by an absence of the large conductance MscL), NhaP-type K^+^(Na^+^)/H^+^ antiporters (CPA1 family), while the low affinity but rapid uptake Trk family transporter is only encoded by the NP-theta and NP-gamma clades. Additionally, NP-theta, NP-iota, hadopelagic NP-gamma and deep marine NP-alpha lineages encode ArsB family Na^+^/H^+^ antiporters.

**Motility and attachment**

Only the NP-delta bins encode a complete repertoire for archaellum assembly and chemotaxis, while both NP-delta and NP-theta clades encode putative Type IV pili assembly clusters (Fig 4, 5, S4, Tables S2, S4). The absence of an archaellum in the clades most adapted to this habitat is not surprising, as this is one of the most sensitive apparatuses and processes to high hydrostatic pressure, while also extremely energy demanding and therefore rarely occurring in sediment lineages [36, 45, 46]. Conversely, the capacity for attachment indicated by the Type IV pili could provide the possibility to adhere to energy-rich particles, and has been implicated in starvation survival strategies [36]. It would appear therefore that active migration (at a huge energy cost) in the sediment column would be an option only for NP-delta, while NP-theta representatives are well adapted to a sedentary lifestyle.

**Information processing systems, DNA and protein repair**

DNA depurination is the most common source of age-induced DNA damage in marine sediments [47, 48]. The main DNA repair systems in the three sediment lineages seem to be double strand break repair (DBSB) via homologous recombination (HR), and base excision repair (BER), while key NER enzymes such as XPD and XPB/Bax1 are missing, as in most *Ca*. Nitrosopumilales & *Ca.* Nitrosotaleales (Tables S2, S4, Fig 4, 5 & S4). While all essential proteins for HR are present in NP-theta and ΝP-iota, NP-delta bins lack a homolog of the Hef helicase domain protein involved in repair of stalled replication forks [49]. However, functional redundancy between Hef and Hjc (Holliday junction resolvase), present in all AOA, has been observed in *H. volcanii*, ensuring that all sediment bins are able to process Holliday junctions and restart collapsed replication forks [50].

The arsenal of monofunctional and bifunctional DNA glycosylases and endonucleases involved in base excision repair (BER) [51], the pathway responsible for the repair of modified (oxidized, alkylated or deaminated) or mismatched bases is present, with some differences, in all sediment clades (Fig. 4, 5, S4). For example, while NP-theta bins encode uracil DNA glycosylases of families 4&5 (Udg), a methylpurine/alkyladenine-DNA glycosylase (Mpg), a 3-methyladenine DNA glycosylase (AlkA) and an 8-oxoguanine (8-oxoG) DNA glycosylase (Ogg1), NP-delta bins encode only family 5 Udg and AlkA glycosylases, while NP-iota bins encode family 4 Udg, Mpg and Ogg1 glycosylases as in hadal NP-α lineages [27]. Additionally, they all encode, as all AOA, an EndoIII/Nth homolog, which has both 8-oxoG DNA glycosylase/AP (apurinic or apyrimidinic site) lyase activities. It is plausible that NP-theta bins encoding four different DNA glycosylases are better equipped at recognizing various types of DNA damage than other NP clades. However, it should be noted that while all superfamilies of glycosylases exhibit substrate specificity, e.g. Udg for uracil pairs [52], Ogg1 for 8-oxoG, AlkA and Mpg for methylated and alkylated bases [53], there is a substrate overlap among superfamily members (e.g. among AlkA and Mpg), so in principle all clades have the capability to recognize the basic types of damaged bases.

The UvrABC system of NER [54, 55], present in AOA, is present only in NP-delta and one NP-theta bin and is also absent from the hadalopelagic and deep marine NP representatives (Fig. 4, 5, Tables S2, S4). Interestingly, the Uvr system has been implicated in transcription-coupled repair in halophilic archaea [56].

While all clades encode the single subunit family B polymerase PolB1, no family D polymerase subunits (DP1, DP2) were detected in NP-iota (Fig. 5, S4). The absence of PolD is also observed in hadal lineages belonging to the NP-alpha clade (erroneously referred to as PolB by [27]) as well as Ca. *Nitrosocaldales* [11, 57]. This provides even stronger support to the hypothesis that PolB1 is the replicative polymerase in Thaumarchaea, performing both leading and lagging strand synthesis as in *S. solfataricus* [58]. This would indicate that PolD in those Thaumarchaea where present has a secondary role in repair, in reversal to the situation observed in Euryarchaeota where PolD is the replicative polymerase while the recognition and complete inhibition by deaminated bases of PolB (PolB3 group) implicated it in repair pathways [59–61]. In the absence of any functional data for the thaumarchaeal polymerases, it is unclear what role PolD plays in repair and what kind of impediment is caused by its absence. Data from euryarchaeal homologs however indicate that this family also stalls upon encountering deaminated bases and can inhibit BER, and could therefore participate in replication-associated repair [59].

NP-theta and NP-iota bins encode a protein-L-isoaspartate carboxylmethyltransferase homolog (pcm), responsible for the repair of D-aspartyl residues in proteins, also found in NP-gamma and NP-eta clades (Fig. 4, 5)[41]. Spontaneous aminoacid racemization is one the most important causes of protein damage in the subsurface marine environments due to the low turnover rates [62], indicating an energy investment towards cell maintenance.

**Supplementary Methods Information**

**Porewater geochemistry of Pacific sediment samples**

Sample processing of Pacific sediment cores: Upon recovery on board, sediment cores were kept in a cold room (4ºC) prior to sample processing. Overlying water was gently sampled and filtrated for geochemical analyses, and then sediment cores were sliced horizontally into 1 to 5cm thickness of sediments. For porewater extraction, sediment samples were immediately centrifuged at 2600 g for 5 min. Extracted porewater were filtered with a 0.45-µm membrane filter and stored at -20° C. Samples for molecular analyses were stored at -80ºC. Porewater geochemistry of the Pacific sediment samples is described in Supplementary Information.

Dissolved oxygen (DO) concentrations in the sediment cores were measured onboard immediately after core recovery using a planar optode oxygen sensor Fibox 3 (PreSens, Regensburg, Germany). The sensor spots were attached to the inside of the transparent polycarbonate core liner tube and oxygen concentrations were measured from the outside [63]. The sensors were calibrated with air-saturated and oxygen-free seawater before measurements.

Nutrient concentrations were measured with a continuous-flow analyser (BL-Tech QUAATRO 2-HR system) [64] onboard. The precision of the phosphate, nitrate, nitrite, and ammonium measurements, based on duplicate measurements, was ± 0.17%, ± 0.17%, ± 0.16%, and ± 0.38%, respectively. When a nutrient concentration exceeded the calibration range, the filtrated porewater was diluted with nutrient-free seawater and were measured again to reduce the concentration to within the calibration range.

**DNA extraction, library construction and sequencing**

DNA for metagenomic sequencing from the Atlantic sediment samples was extracted from ~7 g sediment (~0.7 g sediment in 10 individual lysis tubes) using PowerLyze Soil DNA Isolation Kit (MoBio Laboratories) following the manufacturer’s instructions, except for the following minor modification: the lysing tubes were incubated in water bath of 60^o^C for 15 min prior to beading beating at the highest speed (grade of 6) for 45 seconds on the MP machine. The DNA extracts were iteratively eluted from the 10 spin columns into 100 µL of ddH_2_O for further analysis.

DNA was sheared into 400 bp fragments using Covaris, and libraries were constructed using a Nextera DNA Flex Library Prep kit (Illumina). Metagenomic libraries were sequenced (2×150 bp paired-end) by an Illumina Hiseq 2500 sequencer at the Vienna Biocenter Core Facilities GmbH (Vienna, Austria).

DNA extraction, purification and shotgun metagenomic library construction from the Pacific sediment samples were conducted as described previously [3]. Briefly, from approximately 5 g of the frozen sediment DNA was extracted using DNeasy PowerMax Soil Kit (QIAGEN) and further purified with DNA Clean up MagExtractorTM –PCR & Gel Clean up- (TOYOBO). Then, shotgun metagenomic libraries were constructed using KAPA Hyper Prep Kit from 1 ng DNA or less. The metagenomic sequence libraries were analyzed using Illumina HiSeq2500 with rapid mode (250 bp paired-end).

**Assembly and genome binning of Atlantic sediments**

The sequencing data were processed with Trimmomatic v.0.36 [65] to remove illumina adapters and low quality reads (“SLIDINGWINDOW:10:25”). The quality-controlled reads from the eight samples were de novo co-assembled into contigs using Megahit v.1.1.2 [66] with the k-mer length varying from 27 to 117. Contigs larger than 1000 bp were into automatically binned with MaxBin2 v2.2.5 [67] using the default parameters. The quality of the obtained genome bins was assessed using CheckM v.1.0.7 [68] with the option “lineage_wf”, which uses lineage-specific sets of single-copy genes to estimate completeness and contamination and assigns contamination to strain heterogeneity if amino acid identity is >90%. Genome bins of >50% completeness were manually refined using the gbtools [69] based on the GC content, taxonomic assignments, and differential coverages in different samples. Coverages of contigs in each sample were determined by mapping trimmed reads onto the contigs using BBMap v.37.61 [70]. Taxonomy of contigs were assigned according to the taxonomy of the single-copy marker genes in contigs identified using a script modified from blobology [71] and classified by BLASTn. SSU rRNA sequences in contigs were identified using Barrnap (Seeman 2015, Github), and classified using VSEARCH with the SILVA 132 release [72] as the reference.

To improve the quality of the Thaumarchaeota genomes, we recruited reads from highest-abundance-sample (i.e. highest genome coverage) using BBMap as described above, and the recruited reads were re-assembled using SPAdes v.3.12.0 [73]. After removal of contigs shorter than 1 kb, the resulting scaffolds were visualized and re-binned muanlly using gbtools [69] as described above. The quality of the resulting Thaumarchaeota genomes were checked using the CheckM v.1.0.7 “lineage_wf” command again, based on the Thaumarchaoeta marker gene set (automatically selected by CheckM).

**Metagenomic assembly and binning of Pacific samples**

The reads of the 6 metagenomic samples from deep marine sediments of the Pacific Ocean were quality trimmed and illumina adapters were removed using Trimmomatic [65]⁠. Low-complexity homopolymeric reads (sequences with more than 80% of a single nucleotide) were removed with PRINSEQ [74]⁠. The quality trimmed reads of each metagenomic sample were assembled independently using MEGAHIT [66]⁠. The trimmed reads of all Pacific metagenomes were then mapped back onto the 6 different assemblies using the bowtie2 tool [75]⁠.

The contigs of the six metagenomes were binned with CONCOCT [76]⁠, MetaBAT [77]⁠ and MaxBin 2 [67]⁠ followed by contig dereplication and binning optimization using the DAS tool [78]⁠. Completeness and contamination of bins were evaluated through single copy-marker gene comparison with CheckM (same parameters as above) [68]. Prodigal [79]⁠ was employed with default parameters to predict proteins of optimized bins.

**Phylogeny of *amo*A sequences**

The nucleotide *amo*A sequences of the MAGs reported in this study were retrieved via BLASTN searches (E-value 10^-10^) using the *amo*A sequence of *Nitrosopumilus ureiphilus* (KX950756.1). The *amo*A sequences were incorporated into the curated alignment of *amo*A genes reported by [80],⁠ using MAFFT v7 (“--add” parameter) [81]⁠ followed by the manual inspection of the alignment.

The maximum likelihood phylogenetic tree of *amo*A sequences was reconstructed using IQTREE (v2.0-rc1) [82]⁠ with a GTR+F+I+G model of sequence evolution using a constrained tree search (“-g” parameter) based on the phylogenetic tree reported in [80], and 1,000 ultrafast bootstrap replicates⁠.

**Annotation and comparative genomics analysis**

In addition to the 11 AOA MAGs reported in this work (Table 1), we downloaded 18 completely sequenced AOA genomes, 13 nearly complete genomes and 43 metagenome-assembled or single-amplified genomes from NCBI or IMG database (date: December 2019). In total, our genome collection was composed of 85 genomes (77 AOA plus 8 non-AOA Thaumarchaeota). The complete list of genomes is provided in Table S1. All MAGs and SAGs collected from public databases are more than 70 % complete and have less than 5% contamination, except for Marine Group I thaumarchaeote SCGC RSA3 and Marine Group I thaumarchaeote SCGC AB-629-I23 which are more than 90 % complete and have less than 10 % contamination. Prodigal [79]⁠ was used with default parameters to predict protein sequences when this information was not provided. Annotation of the MAGs assembled in this study was performed automatically using the Microscope annotation platform from Genoscope [83], followed by extensive manual curation.

**ANI comparisons**

Pairwise average nucleotide identity comparisons between MAGs were performed using the ANI script from the enveomics collection [84].

**Sequence alignment**

Primary sequences were aligned with Mafft [85] and visualized with BOXSHADE (ExPASy).

**Data availability**

Raw reads from metagenomic sequencing as well as 16S and *amo*A amplicons have been submitted to NCBI under project accession numbers PRJNA489438, PRJNA529480 (Atlantic samples) and PRJDB9793 (Pacific samples). The Whole Genome Shotgun projects have been deposited at DDBJ/ENA/GenBank under the accession numbers JADNRU000000000, JAEHKP000000000, JAEHKP000000000, JAEHKU000000000, JAEHKV000000000, JAEHKQ000000000, JAEHKR000000000 JAEHKS000000000, JAEHKT000000000. The versions described
in this paper are versions XXXXXX010000000. Assemblies of MAGs reported in this study are also available in the Microbial Genome Annotation & Analysis Platform Microscope (https://mage.genoscope.cns.fr/microscope/home/index.php) [83].

**References**

1. Zhao R, Hannisdal B, Mogollon JM, Jørgensen SL. Nitrifier abundance and diversity peak at deep redox transition zones. *Sci Rep* 2019; **9**: 1–12.

2. Nunoura T, Takaki Y, Kazama H, Hirai M, Ashi J, Imachi H, et al. Microbial diversity in deep-sea methane seep sediments presented by SSU rRNA gene tag sequencing. *Microbes Environ* 2012; **27**: 382–390.

3. Hirai M, Nishi S, Tsuda M, Sunamura M, Takaki Y, Nunoura T. Library construction from subnanogram DNA for pelagic sea water and deep-sea sediments. *Microbes Environ* 2017; **32**: ﻿336-343.

4. Zhao R, Dahle H, Ramírez GA, Jørgensen SL. Indigenous ammonia-oxidizing archaea in oxic subseafloor oceanic crust. *mSystems* 2020; **5**.

5. Jain C, Rodriguez-R LM, Phillippy AM, Konstantinidis KT, Aluru S. High throughput ANI analysis of 90K prokaryotic genomes reveals clear species boundaries. *Nat Commun* 2018; **9**: 5114.

6. Konstantinidis KT, Rosselló-Móra R, Amann R. Uncultivated microbes in need of their own taxonomy. *ISME J* 2017; **11**: 2399–2406.

7. Kirkpatrick JB, Walsh EA, D’Hondt S. Microbial selection and survival in subseafloor sediment. *Front Microbiol* 2019; **10**: 956.

8. Petro C, Starnawski P, Schramm A, Kjeldsen K. Microbial community assembly in marine sediments. *Aquat Microb Ecol* 2017; **79**: 177–195.

9. Orcutt BN, Edwards KJ. Life in the Ocean Crust. *Developments in Marine Geology*. 2014. 175–196.

10. Abby SS, Kerou M, Schleper C. Ancestral reconstructions decipher major adaptations of ammonia-oxidizing archaea upon radiation into moderate terrestrial and marine environments. *MBio* 2020; **11**.

11. Abby SS, Melcher M, Kerou M, Krupovic M, Stieglmeier M, Rossel C, et al. *Candidatus* Nitrosocaldus cavascurensis, an ammonia oxidizing, extremely thermophilic archaeon with a highly mobile genome. *Front Microbiol* 2018; **9**: 28.

12. Herbold CW, Lehtovirta-Morley LE, Jung M-Y, Jehmlich N, Hausmann B, Han P, et al. Ammonia-oxidising archaea living at low pH: Insights from comparative genomics. *Environ Microbiol* 2017; **19**: 4939–4952.

13. Spang A, Poehlein A, Offre P, Zumbragel S, Haider S, Rychlik N, et al. The genome of the ammonia-oxidizing *Candidatus* Nitrososphaera gargensis: insights into metabolic versatility and environmental adaptations. *Env Microbiol* 2012; **14**: 3122–3145.

14. Wang B, Qin W, Ren Y, Zhou X, Jung M-Y, Han P, et al. Expansion of Thaumarchaeota habitat range is correlated with horizontal transfer of ATPase operons. *ISME J* 2019; **13**: 3067–3079.

15. LaRowe DE, Burwicz E, Arndt S, Dale AW, Amend JP. Temperature and volume of global marine sediments. *Geology* 2017.

16. Morono Y, Terada T, Nishizawa M, Ito M, Hillion F, Takahata N, et al. Carbon and nitrogen assimilation in deep subseafloor microbial cells. *Proc Natl Acad Sci* 2011; **108**: 18295–18300.

17. Vuillemin A, Wankel SD, Coskun ÖK, Magritsch T, Vargas S, Estes ER, et al. Archaea dominate oxic subseafloor communities over multimillion-year time scales. *Sci Adv* 2019; **5**: eaaw4108.

18. Li M, Baker BJ, Anantharaman K, Jain S, Breier JA, Dick GJ. Genomic and transcriptomic evidence for scavenging of diverse organic compounds by widespread deep-sea archaea. *Nat Commun* 2015; **6**: 8933.

19. Desguin B, Goffin P, Viaene E, Kleerebezem M, Martin-Diaconescu V, Maroney MJ, et al. Lactate racemase is a nickel-dependent enzyme activated by a widespread maturation system. *Nat Commun* 2014; **5**: 3615.

20. Desguin B, Soumillion P, Hausinger RP, Hols P. Unexpected complexity in the lactate racemization system of lactic acid bacteria. *FEMS Microbiol Rev* 2017; **41**: S71–S83.

21. Kerou M, Offre P, Valledor L, Abby SS, Melcher M, Nagler M, et al. Proteomics and comparative genomics of *Nitrososphaera viennensis* reveal the core genome and adaptations of archaeal ammonia oxidizers. *Proc Natl Acad Sci U S A* 2016; **113**: E7937–E7946.

22. Bienert GP, Desguin B, Chaumont F, Hols P. Channel-mediated lactic acid transport: a novel function for aquaglyceroporins in bacteria. *Biochem J* 2013; **454**: 559–570.

23. Desguin B. Lactate Racemization and Beyond. *J Bacteriol Parasitol* 2018; **09**.

24. Roche J, Girard E, Mas C, Madern D. The archaeal LDH-like malate dehydrogenase from *Ignicoccus islandicus* displays dual substrate recognition, hidden allostery and a non-canonical tetrameric oligomeric organization. *J Struct Biol* 2019; **208**: 7–17.

25. Lee B Il, Chang C, Cho SJ, Eom SH, Kim KK, Yu YG, et al. Crystal structure of the MJ0490 gene product of the hyperthermophilic archaebacterium *Methanococcus jannaschii*, a novel member of the lactate/malate family of dehydrogenases. *J Mol Biol* 2001; **307**: 1351–1362.

26. León-zayas R, Novotny M, Podell S, Shepard CM, Berkenpas E, Nikolenko S, et al. Single cells within the puerto rico trench suggest hadal adaptation of microbial lineages. *AEM* 2015; **81**: 8265–8276.

27. Wang Y, Huang J-M, Cui G-J, Nunoura T, Takaki Y, Li W-L, et al. Genomics insights into ecotype formation of ammonia-oxidizing archaea in the deep ocean. *Environ Microbiol* 2019; **21**: 716–729.

28. Walker CB, de la Torre JR, Klotz MG, Urakawa H, Pinel N, Arp DJ, et al. *Nitrosopumilus maritimus* genome reveals unique mechanisms for nitrification and autotrophy in globally distributed marine crenarchaea. *Proc Natl Acad Sci U S A* 2010; **107**: 8818–8823.

29. Simon J, Klotz MG. Diversity and evolution of bioenergetic systems involved in microbial nitrogen compound transformations. *Biochim Biophys Acta - Bioenerg* 2013; **1827**: 114–135.

30. Chadwick GL, Hemp J, Fischer WW, Orphan VJ. Convergent evolution of unusual complex I homologs with increased proton pumping capacity: energetic and ecological implications. *ISME J* 2018; **12**: 2668–2680.

31. Klotz MG, Arp DJ, Chain PS, El-Sheikh AF, Hauser LJ, Hommes NG, et al. Complete genome sequence of the marine, chemolithoautotrophic, ammonia-oxidizing bacterium *Nitrosococcus oceani* ATCC 19707. *Appl Environ Microbiol* 2006; **72**: 6299–6315.

32. Norton JM, Klotz MG, Stein LY, Arp DJ, Bottomley PJ, Chain PSG, et al. Complete genome sequence of *Nitrosospira multiformis*, an ammonia-oxidizing bacterium from the soil environment. *Appl Environ Microbiol* 2008; **74**: 3559–72.

33. Ohke Y, Sakoda A, Kato C, Sambongi Y, Kawamoto J, Kurihara T, et al. Regulation of cytochrome c - and quinol oxidases, and piezotolerance of their activities in the deep-sea piezophile *Shewanella violacea* DSS12 in response to growth conditions. *Biosci Biotechnol Biochem* 2013; **77**: 1522–1528.

34. Empadinhas N, Da Costa MS. Osmoadaptation mechanisms in prokaryotes: Distribution of compatible solutes. *Int Microbiol* . 2008.

35. Vannier P, Michoud G, Oger P, Marteinsson VP, Jebbar M. Genome expression of *Thermococcus barophilus* and *Thermococcus kodakarensis* in response to different hydrostatic pressure conditions. *Res Microbiol* 2015; **166**: 717–725.

36. Lever MA, Rogers KL, Lloyd KG, Overmann J, Schink B, Thauer RK, et al. Life under extreme energy limitation: a synthesis of laboratory- and field-based investigations. *FEMS Microbiol Rev* 2015; **39**: 688–728.

37. Amrani A, Bergon A, Holota H, Tamburini C, Garel M, Ollivier B, et al. Transcriptomics reveal several gene expression patterns in the piezophile desulfovibrio hydrothermalis in response to hydrostatic pressure. *PLoS One* 2014; **9**: e106831.

38. Amrani A, van Helden J, Bergon A, Aouane A, Ben Hania W, Tamburini C, et al. Deciphering the adaptation strategies of *Desulfovibrio piezophilus* to hydrostatic pressure through metabolic and transcriptional analyses. *Environ Microbiol Rep* 2016; **8**: 520–526.

39. Michoud G, Jebbar M. High hydrostatic pressure adaptive strategies in an obligate piezophile *Pyrococcus yayanosii.* *Sci Rep* 2016; **6**: 27289.

40. Watrin L, Martin-Jezequel V, Prieur D. Minimal amino acid requirements of the hyperthermophilic archaeon Pyrococcus abyssi, isolated from deep-sea hydrothermal vents. *Appl Environ Microbiol* 1995; **61**: 2069–2069.

41. Ngugi DK, Blom J, Alam I, Rashid M, Ba-Alawi W, Zhang G, et al. Comparative genomics reveals adaptations of a halotolerant thaumarchaeon in the interfaces of brine pools in the Red Sea. *ISME J* 2015; **9**: 396–411.

42. Offre P, Kerou M, Spang A, Schleper C. Variability of the transporter gene complement in ammonia-oxidizing archaea. *Trends Microbiol* 2014; **22**: 665–675.

43. Santoro AE, Dupont CL, Richter RA, Craig MT, Carini P, McIlvin MR, et al. Genomic and proteomic characterization of ‘*Candidatus* Nitrosopelagicus brevis’: An ammonia-oxidizing archaeon from the open ocean. *Proc Natl Acad Sci U S A* 2015; **112**: 1173–8.

44. Bayer B, Vojvoda J, Offre P, Alves RJ, Elisabeth NH, Garcia JA, et al. Physiological and genomic characterization of two novel marine thaumarchaeal strains indicates niche differentiation. *ISME J* 2016; **10**: 1051–1063.

45. Bartlett DH. Pressure effects on in vivo microbial processes. *Biochim Biophys Acta - Protein Struct Mol Enzymol* 2002; **1595**: 367–381.

46. Tully BJ, Heidelberg JF. Potential mechanisms for microbial energy acquisition in oxic deep sea sediments. *Appl Environ Microbiol* 2016; **82**: ﻿4232-4243.

47. Hoehler TM, Jørgensen BB. Microbial life under extreme energy limitation. *Nat Rev Microbiol* 2013; **11**: 83–94.

48. Jørgensen BB, Marshall IPG. Slow microbial life in the seabed. *Ann Rev Mar Sci* 2016; **8**: 311–332.

49. Ishino Y, Narumi I. DNA repair in hyperthermophilic and hyperradioresistant microorganisms. *Curr Opin Microbiol* 2015; **25**: 103–112.

50. White MF, Allers T. DNA repair in the archaea—an emerging picture. *FEMS Microbiol Rev* 2018.

51. Grasso S, Tell G. Base excision repair in Archaea: back to the future in DNA repair. *DNA Repair* 2014; **21**: 148–157.

52. Lucas-Lledo JI, Maddamsetti R, Lynch M. Phylogenomic analysis of the uracil-DNA glycosylase superfamily. *Mol Biol Evol* 2011; **28**: 1307–1317.

53. Denver DR. An Evolutionary analysis of the helix-hairpin-helix superfamily of DNA repair glycosylases. *Mol Biol Evol* 2003; **20**: 1603–1611.

54. Morita R, Nakane S, Shimada A, Inoue M, Iino H, Wakamatsu T, et al. Molecular mechanisms of the whole dna repair system: a comparison of bacterial and eukaryotic systems. *J Nucleic Acids* 2010; **2010**: 1–32.

55. Truglio JJ, Croteau DL, Van Houten B, Kisker C. Prokaryotic nucleotide excision repair: The UvrABC system. *Chem Rev* 2006; **106**: 233–252.

56. Stantial N, Dumpe J, Pietrosimone K, Baltazar F, Crowley DJ. Transcription-coupled repair of UV damage in the halophilic archaea. *DNA Repair (Amst)* 2016; **41**: 63–68.

57. Daebeler A, Herbold CW, Vierheilig J, Sedlacek CJ, Pjevac P, Albertsen M, et al. Cultivation and genomic analysis of “*Candidatus* Nitrosocaldus islandicus,” an obligately thermophilic, ammonia-oxidizing thaumarchaeon from a hot spring biofilm in Graendalur Valley, Iceland. *Front Microbiol* 2018; **9**.

58. Yan J, Beattie TR, Rojas AL, Schermerhorn K, Gristwood T, Trinidad JC, et al. Identification and characterization of a heterotrimeric archaeal DNA polymerase holoenzyme. *Nat Commun* 2017; **8**: 15075.

59. Abellón-Ruiz J, Ishino S, Ishino Y, Connolly BA. Archaeal DNA Polymerase-B as a DNA template guardian: links between polymerases and base/alternative excision repair enzymes in handling the deaminated bases uracil and hypoxanthine. *Archaea* 2016; **2016**: 1–8.

60. Kushida T, Narumi I, Ishino S, Ishino Y, Fujiwara S, Imanaka T, et al. Pol B, a Family B DNA polymerase, in *Thermococcus kodakarensis* is important for DNA repair, but not DNA replication. *Microbes Environ* 2019; **34**: 316–326.

61. Makarova KS, Krupovic M, Koonin E V. Evolution of replicative DNA polymerases in archaea and their contributions to the eukaryotic replication machinery. *Front Microbiol* 2014; **5**: 354.

62. Mhatre SS, Kaufmann S, Marshall IPG, Obrochta S, Andrèn T, Jørgensen BB, et al. Microbial biomass turnover times and clues to cellular protein repair in energy-limited deep Baltic Sea sediments. *FEMS Microbiol Ecol* 2019.

63. Hiraoka S, Hirai M, Matsui Y, Makabe A, Minegishi H, Tsuda M, et al. Microbial community and geochemical analyses of trans-trench sediments for understanding the roles of hadal environments. *ISME J* 2020; **14**: 740–756.

64. Nomaki H, Arai K, Suga H, Toyofuku T, Wakita M, Nunoura T, et al. Sedimentary organic matter contents and porewater chemistry at upper bathyal depths influenced by the 2011 off the Pacific coast of Tohoku Earthquake and tsunami. *J Oceanogr* 2016; **72**: 99–111.

65. Bolger AM, Lohse M, Usadel B. Trimmomatic: a flexible trimmer for Illumina sequence data. *Bioinformatics* 2014; **30**: 2114–2120.

66. Li D, Liu C-M, Luo R, Sadakane K, Lam T-W. MEGAHIT: an ultra-fast single-node solution for large and complex metagenomics assembly via succinct de Bruijn graph. *Bioinformatics* 2015; **31**: 1674–1676.

67. Wu Y-W, Simmons BA, Singer SW. MaxBin 2.0: an automated binning algorithm to recover genomes from multiple metagenomic datasets. *Bioinformatics* 2016; **32**: 605–607.

68. Parks DH, Imelfort M, Skennerton CT, Hugenholtz P, Tyson GW. CheckM: assessing the quality of microbial genomes recovered from isolates, single cells, and metagenomes. *Genome Res* 2015; **25**: 1043–1055.

69. Seah BKB, Gruber-Vodicka HR. gbtools: Interactive visualization of metagenome bins in R. *Front Microbiol* 2015; **6**.

70. Bushnell B. BBMap: a fast, accurate, splice-aware aligner. *Joint Genome Instritute, department of energy* . 2014.

71. Kumar S, Jones M, Koutsovoulos G, Clarke M, Blaxter M. Blobology: exploring raw genome data for contaminants, symbionts and parasites using taxon-annotated GC-coverage plots. *Front Genet* 2013; **4**.

72. Quast C, Pruesse E, Yilmaz P, Gerken J, Schweer T, Yarza P, et al. The SILVA ribosomal RNA gene database project: improved data processing and web-based tools. *Nucleic Acids Res* 2012; **41**: D590–D596.

73. Bankevich A, Nurk S, Antipov D, Gurevich AA, Dvorkin M, Kulikov AS, et al. SPAdes: A new genome assembly algorithm and its applications to single-cell sequencing. *J Comput Biol* 2012; **19**: 455–477.

74. Schmieder R, Edwards R. Quality control and preprocessing of metagenomic datasets. *Bioinformatics* 2011; **27**: 863–864.

75. Langmead B, Salzberg SL. Fast gapped-read alignment with Bowtie 2. *Nat Methods* 2012.

76. Alneberg J, Bjarnason BS, de Bruijn I, Schirmer M, Quick J, Ijaz UZ, et al. Binning metagenomic contigs by coverage and composition. *Nat Methods* 2014; **11**: 1144–1146.

77. Kang DD, Froula J, Egan R, Wang Z. MetaBAT, an efficient tool for accurately reconstructing single genomes from complex microbial communities. *PeerJ* 2015; **3**: e1165.

78. Sieber CMK, Probst AJ, Sharrar A, Thomas BC, Hess M, Tringe SG, et al. Recovery of genomes from metagenomes via a dereplication, aggregation and scoring strategy. *Nat Microbiol* 2018; **3**: 836–843.

79. Hyatt D, Chen GL, LoCascio PF, Land ML, Larimer FW, Hauser LJ. Prodigal: Prokaryotic gene recognition and translation initiation site identification. *BMC Bioinformatics* 2010.

80. Alves RJE, Minh BQ, Urich T, von Haeseler A, Schleper C. Unifying the global phylogeny and environmental distribution of ammonia-oxidising archaea based on amoA genes. *Nat Commun* 2018; **9**: 1517.

81. Katoh K, Standley DM. MAFFT multiple sequence alignment software version 7: improvements in performance and usability. *Mol Biol Evol* 2013; **30**: 772–780.

82. Minh BQ, Schmidt HA, Chernomor O, Schrempf D, Woodhams MD, von Haeseler A, et al. IQ-TREE 2: new models and efficient methods for phylogenetic inference in the genomic era. *Mol Biol Evol* 2020; **37**: 1530–1534.

83. Vallenet D, Calteau A, Cruveiller S, Gachet M, Lajus A, Josso A, et al. MicroScope in 2017: an expanding and evolving integrated resource for community expertise of microbial genomes. *Nucleic Acids Res* 2017; **45**: D517–D528.

84. Rodriguez-R L, Konstantinidis K. The enveomics collection: a toolbox for specialized analyses of microbial genomes and metagenomes. 2016.

85. Kazutaka Katoh DMS. MAFFT multiple sequence alignment software version 7: improvements in performance and usability. *Mol Biol Evol* 2013; **30**: 772.

**Supplementary figure legends**

**Supplementary figure 1 (S1).** Porewater profiles of oxygen and nitrate in the sediment cores used in this study. In the two Pacific cores (YK1309-1N and YK1312-12N), the dashed lines denote the sediment-water interface. Note in the two Atlantic cores the sediment-water interfaces were not properly recovered by the piston/gravity coring. Different axis scales were used for different cores. Data of NP-U1383E were from [12], and GS14-GC08 from [39].

**Supplementary figure 2a (S2a) Phylogenomic tree of AOA and non-AOA Thaumarchaeota.**

The phylogenomic tree was reconstructed based on the concatenated alignment of 79 markers comprising 17,483 amino acid sites using a maximum likelihood approach (see Materials and methods). Yellow circles represent 100 % bootstrap support of nodes. The metagenome-assembled genomes (MAGs) reported in this study are shown in bold. Representative genomes of NP-subclades were added for subclade clarification. Information about the ecological distribution of AOA clades is provided. The scale bar indicates the number of substitutions per amino acid site. In most cases congruence between *amo*A-based and 16S rRNA-based clades was inferred from complete genomes or MAGs where both genes were present [14, 22, 23]. For other cases: 1) *amo*A-NP-iota/16S-NP-lambda pair: the congruence of these two clades is inferred from phylogenetic trees and congruence in relative abundances (Zhao and Jorgensen, unpublished data) of sediment layers with high and changing relative abundances; 2) *amo*A*-*NP-delta/16S-NP-epsilon,-zeta pair: The congruence of these two clades is based on the MAG of CSP1 (with both genes), the close relationship of 16S-NP-epsilon with CSP1 and the occurrence pattern of this clade in marine sediments (see [12], and data not shown); and 3) *amo*A-NP-theta/16S-NP-alpha,-eta,-upsilon pair: congruence based on [14], own phylogenetic reconstructions (unpublished data) and relative abundances in marine sediment layers. Abbreviations: NA; not assigned.

**(S2b).** Pairwise average nucleotide identity calculations among the deep marine sediment-derived MAGs. The archaeon CSP1 MAG was included in the analyses to have all the representatives of the NP-delta clade.

**Supplementary figure 3 (S3).** Extended heatmap depicting the distribution and abundance of genes involved in the main functional categories discussed in the text. Abbreviations: nit2, nitrilase/omega-amidase; ureA, urease subunit gamma; mco1, multicopper oxidase family 1; fdh, formate dehydrogenase; larA, lactate racemase; pgi, phosphoglucose isomerase; proDH, proline dehydrogenase; rocA, 1-pyrroline-5-carboxylate dehydrogenase; oat, putative ornithine--oxo-glutarate aminotransferase/class III aminotransferase; ggt, ﻿g-glutamyl transpeptidase; kal, 3-aminobutyryl-CoA ammonia lyase; kat, putative 3-aminobutyryl-CoA aminotransferase; argD, acetylornithine transaminase; serC, serine-puryvate aminotransferase; ilvA, threonine/serine ammonia-lyase; ilvE, branched-chain-amino-acid transaminase; aspC, aspartate/tyrosine/aromatic aminotransferase; gvtTPH, glycine cleavage system proteins T/P/H; metH, methionine synthase II (cobalamin-independent); metE, methionine synthase I (cobalamin-dependent); tbp, TATA-box binding protein; rpoS54, AAA family ATPase/RNA polymerase sigma factor 54 interaction domain; phr, photolyase; polD2, DNA polymerase D, large subunit DP2; uvrABC, the Uvr excision repair system endonucleases ABC; hef, Hef/FANCM/Mph1-like helicase; udg4/5, Uracil DNA glycosylase family 4/5; mpg, methylpurine/alkyladenine-DNA glycosylase; ogg1, 8-oxoguanine DNA glycosylase; alkA, DNA-3-methyladenine glycosylase; tag, 3-methyladenine DNA glycosylase; endoV, endonuclease V; POP4, RNase P/RNase MRP subunit p29, uspA, universal stress protein A; pcm, protein-L-isoaspartate carboxylmethyltransferase; ipct/dipps, bifunctional CTP:inositol-1-phosphate cytidylyltransferase/di-myo-inositol-1,3′-phosphate-1′-phosphate synthase; cspC, cold-shock protein A; cshA, cold-shock DEAD-box protein A; LLM, luciferase-like monooxygenase family protein; nanM, N-acetylneuraminic acid mutarotase; flaK, archaeal preflagellin peptidase FlaK; cheY, chemotaxis response regulator CheY; cheAB, chemotactic sensor histidine kinase cheA & methylesterase cheB; XerD/XerC family integrases; Protease and transporter classes can be found in Table S4. All locus tags and cluster information are in Supplementary tables S2 & S4.

**Supplementary figure 4 (S4).** ML Phylogenetic tree of 2HADH sequences.

A dehydrogenase protein cluster comprising 14 sequences (colored in blue in the phylogenetic tree) was added using MAFFT v7 [122]⁠ (“mafft-linsi –add”) to the structure-based reference alignment of 2HADH utilized by [77].⁠ The alignment was trimmed in trimmAl⁠ (“trimal -gt 0.2”) and a ML phylogenetic tree was calculated using IQTREE (v2.0-rc1) [57] with a GTR+F+I+G model and 1,000 ultrafast bootstrap replicates⁠. Yellow circles represent nodes with 100 % bootstrap support. The scale bar indicates the number of amino acid substitutions per site.

**Supplementary figure 5 (S5).** Sequence alignment of lactate dehydrogenase (LDH) and malate dehydrogenase (MDH) homologs, shaded in yellow and green, respectively. Conserved residues are shaded in black and grey. The universally conserved substrate binding Arg171 in the LDH/MDH superfamily is indicated in red. Residues important for substrate discrimination and active site architecture are shaded in orange and discussed in the text. The residue determining the cofactor specificity is shaded in green. Residue numbering refers to the LDH numbering as in [82].

Primary sequences from the following organisms were used to generate the alignment (Uniprot accession numbers in parentheses): Gster, *Geobacillus stearothermophilus* (P00344); Tth, *Thermus thermophilus* (Q5SJA1); Blon, *Bifidobacterium longum* (E8ME30); Ctep, *Chlorobaculum tepidum* (P80039); Nvie, *Nitrososphaera viennensis* (A0A060HG74); Nmar, *Nitrosopumilus maritimus* (A9A450); Nkor, *Nitrosarchaeum koreense* (F9CUM5); Nbrev, *Ca.* Nitrosopelagicus brevis (A0A0A7V4F4); Nuzo, *Ca.* Nitrosotenuis uzonensis (V6AR53); Mjan, *Methanocaldococcus jannaschii* (Q60176); Iisl, *Ignicoccus islandicus* (A0A0U3FQH7); Msed, *Metallosphaera sedula* (A4YDY0). Sequences from the marine sediment AOA MAGs reported in this study are in bold and their locus tags can be found in Table S4.

**Supplementary figure 6 (S6).** Phylogenetic tree of lactate racemase sequences. A total of 1813 proteins of the PF09861 superfamily⁠, 19 LarA sequences reported by [81]⁠ (colored in blue) and 7 putative LarA sequences identified in the MAGs reported in this study (colored in red) were aligned using MAFFT v7 (FFT-NS-2 strategy)[123]⁠. The alignment output was used to construct an approximately-maximum-likelihood phylogenetic tree using FastTree v2.1.11 with default parameters [124]⁠.

**Description of Supplementary Tables**

**Table S1**: List and description of genomes (complete, MAGs and SAGs) used in the study

**Table S2**: Description of protein families generated from the dataset.

**Table S3**: List of archaeal single copy marker genes from Rinke et al. 2013 used in the concatenated alignment for the generation of the phylogenomic tree in Fig. 2.

**Table S4**: Locus tags and associated annotation of central metabolic pathways of the MAGs reported in this study.
