# Supplementary figures and images for: Genomes of Thaumarchaeota from deep sea sediments reveal specific adaptations of three independently evolved lineages
Source: ISME J. 2021 Apr 1;15(9):2792–808. doi: 10.1038/s41396-021-00962-6 (PMC8397731; doi:10.1038/s41396-021-00962-6)

Figure S1

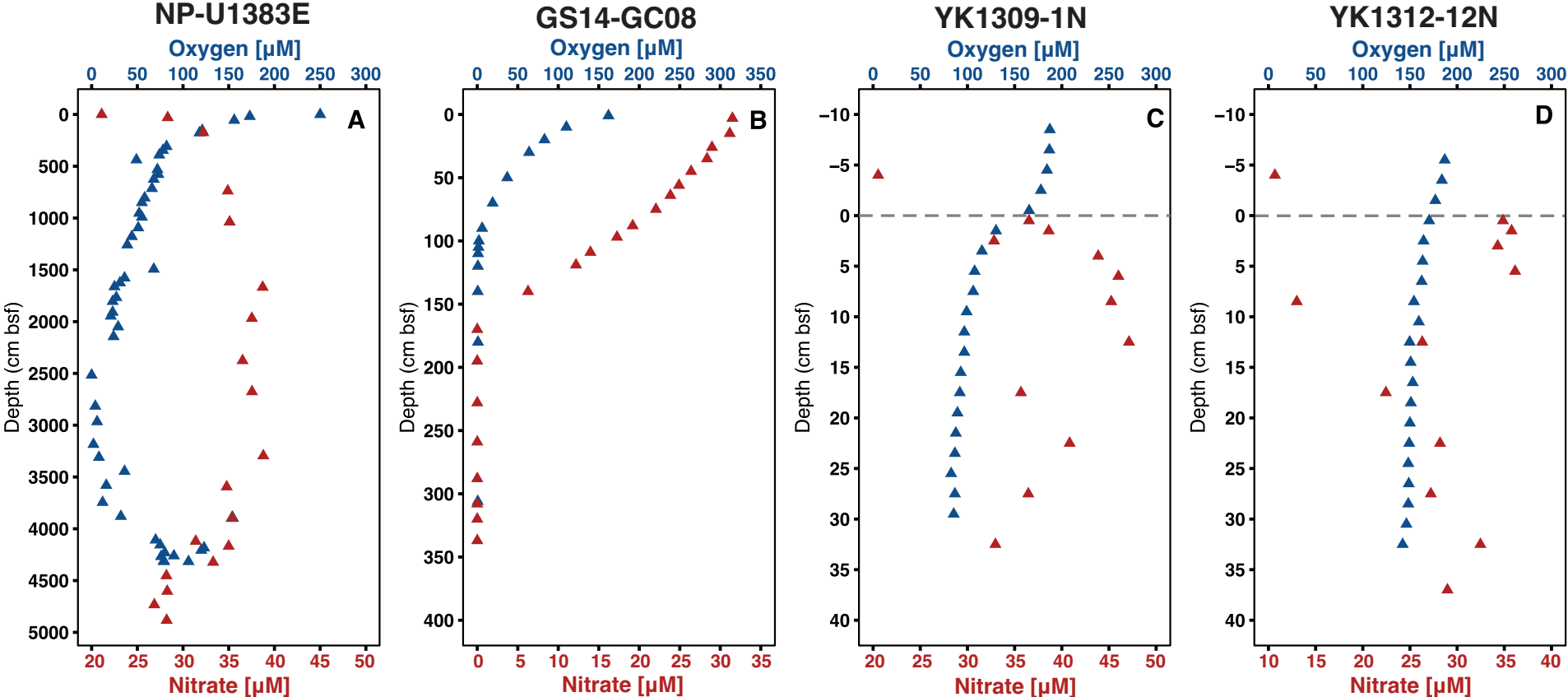

Supplement: Supplementary file 2 — Figure S1 [file 41396_2021_962_MOESM2_ESM.pdf]

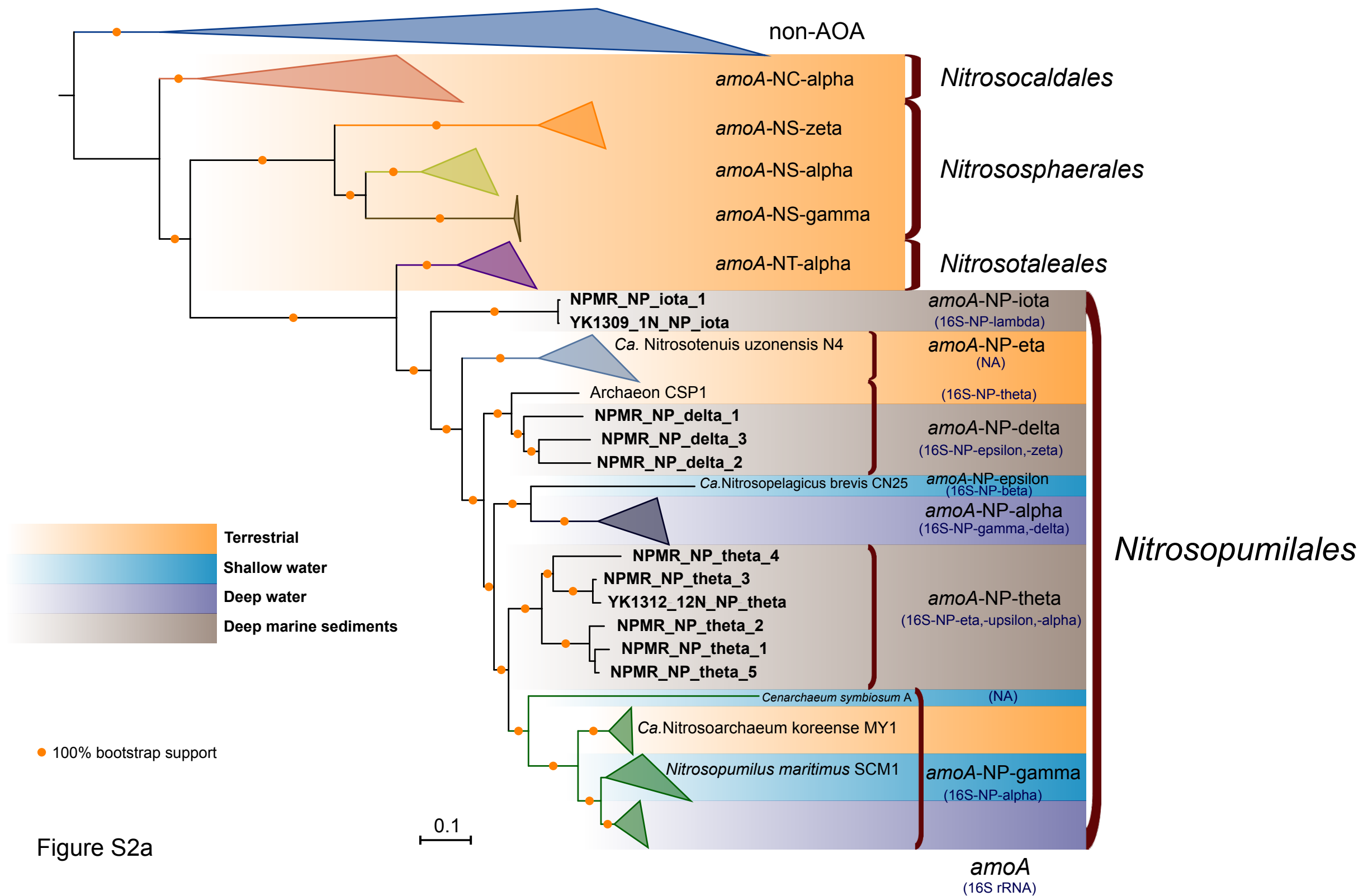

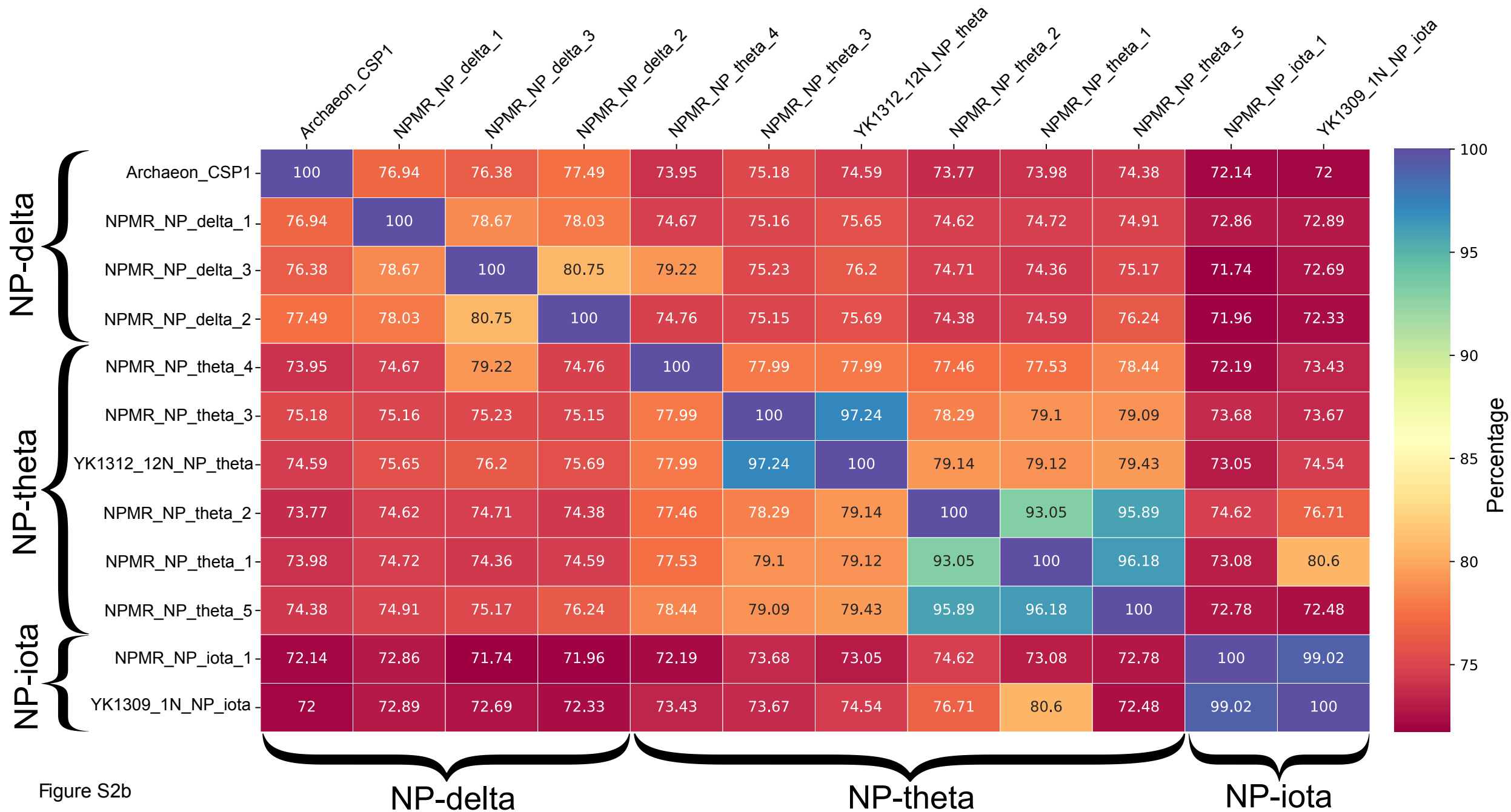

Supplement: Supplementary file 3 — Figure S2 [file 41396_2021_962_MOESM3_ESM.pdf]

Tree scale: 1

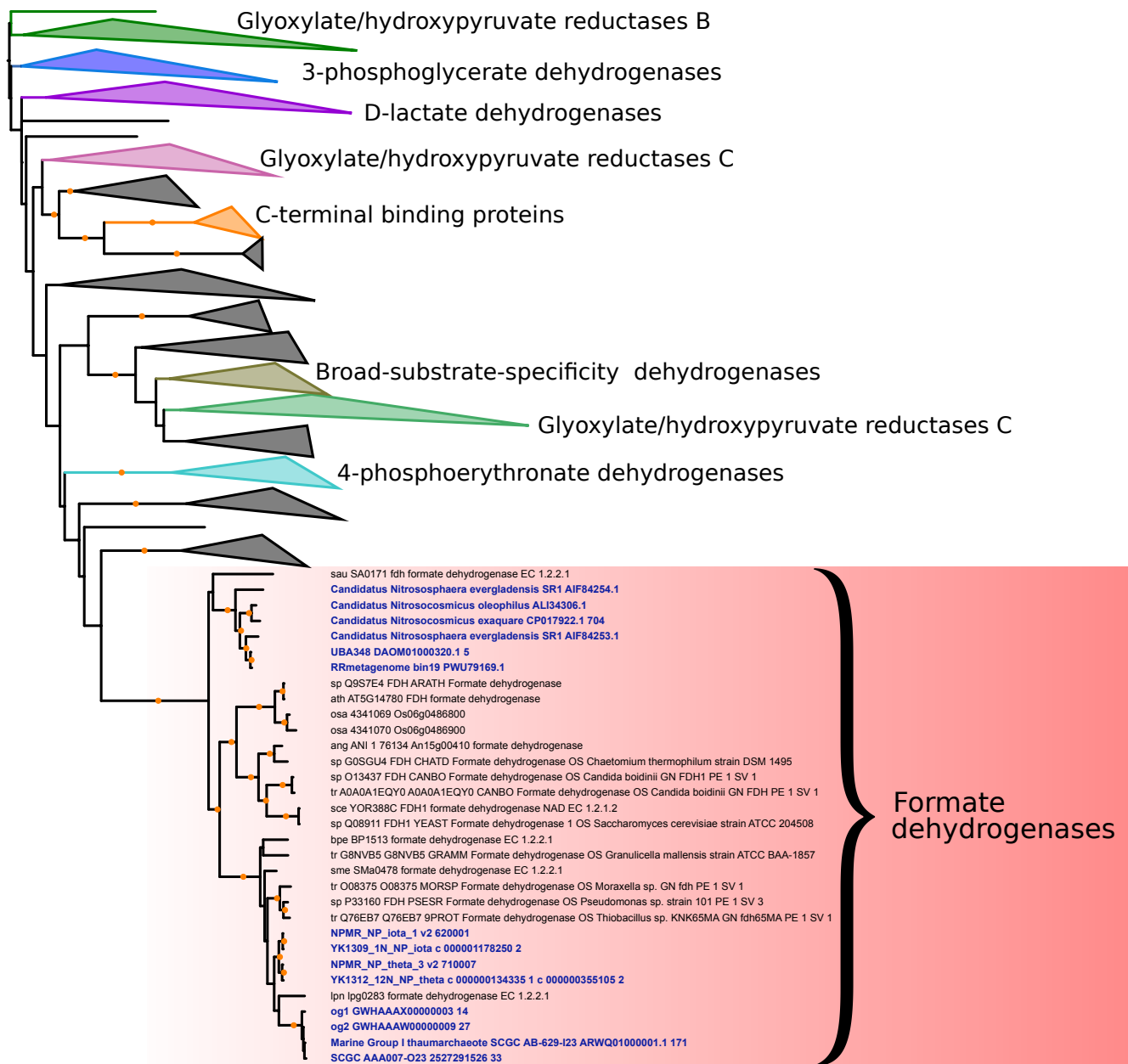

Figure S4

Supplement: Supplementary file 5 — Figure S4 [file 41396_2021_962_MOESM5_ESM.pdf]

---

## Colored ranges

Putative LarA from sediment AOA

 LarA sequences

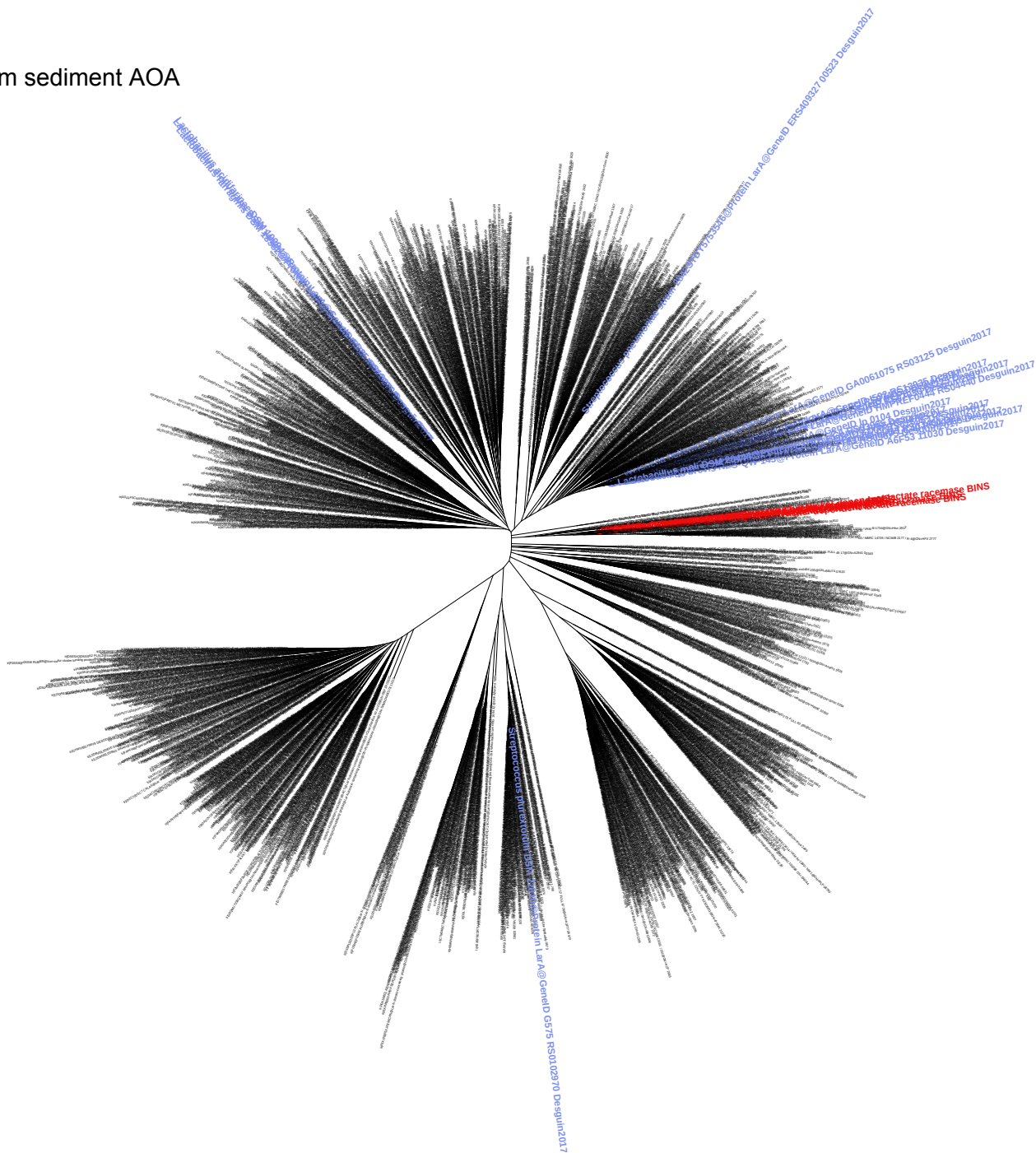

Figure S6

Supplement: Supplementary file 7 — Figure S6 [file 41396_2021_962_MOESM7_ESM.pdf]
