# Supplementary material for: Genomes of Thaumarchaeota from deep sea sediments reveal specific adaptations of three independently evolved lineages
Source: ISME J. 2021 Apr 1;15(9):2792–808. doi: 10.1038/s41396-021-00962-6 (PMC8397731; doi:10.1038/s41396-021-00962-6)
Supplement: Supplementary file 4 — Figure S3 [file 41396_2021_962_MOESM4_ESM.pdf]

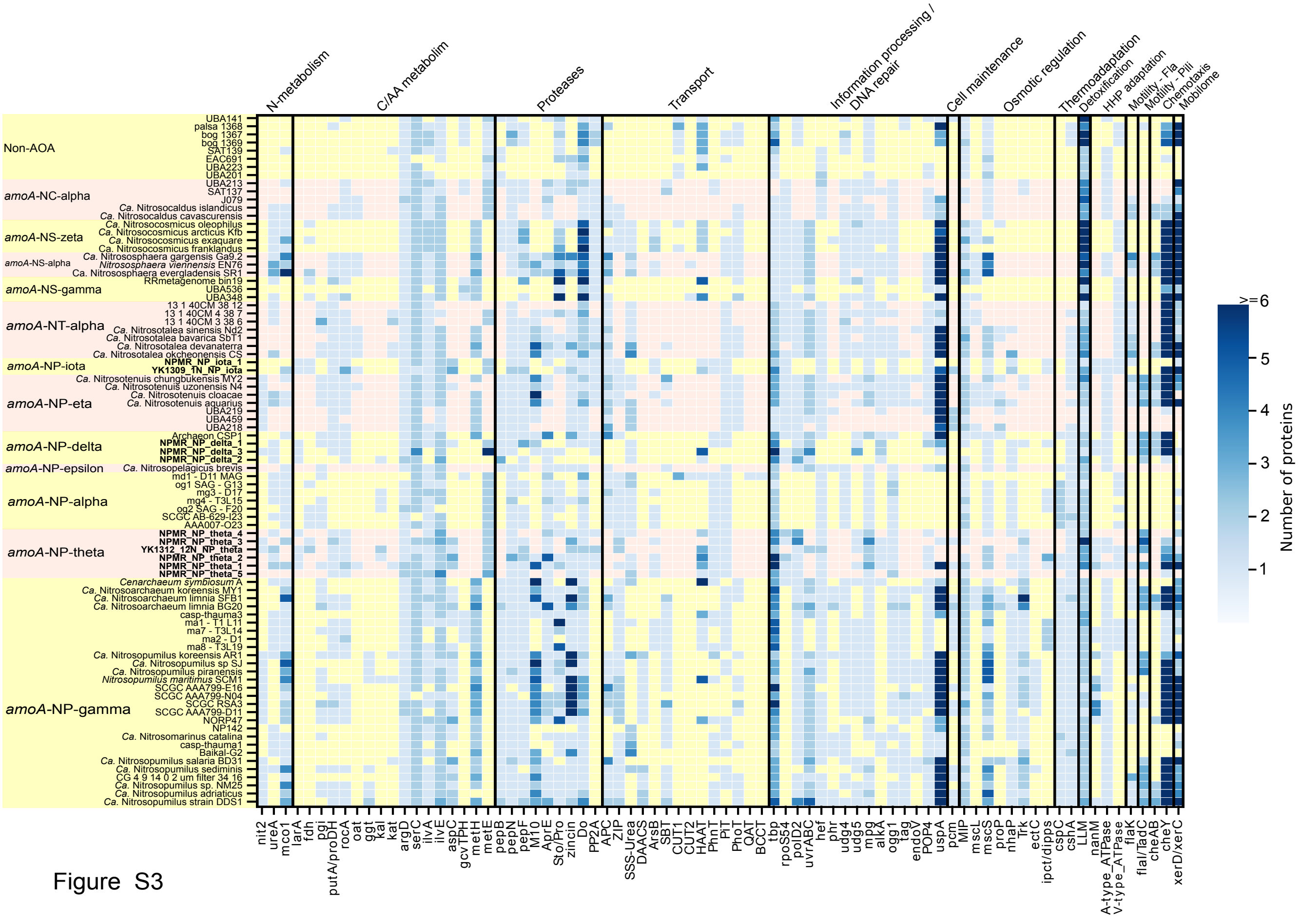
N-metabolism
C/AA metabolim
Proteases
Transport
Information processing / DNA repair
Cell maintenance
Osmotic regulation
Thermoadaptation
Detoxification
HHP adaptation
Motility - Fla
Motility - Pili
Chemotaxis
Mobilome
Non-AOA
amoA-NC-alpha
amoA-NS-zeta
amoA-NS-alpha
amoA-NS-gamma
amoA-NT-alpha
amoA-NP-iota
amoA-NP-eta
amoA-NP-delta
amoA-NP-epsilon
amoA-NP-alpha
amoA-NP-theta
amoA-NP-gamma
UBA141
palsa 1368
bog 1367
bog 1369
SAT139
EAC691
UBA223
UBA201
UBA213
SAT137
J079
Ca. Nitrosocaldus islandicus
Ca. Nitrosocaldus cavascurensis
Ca. Nitrosocosmicus oleophilus
Ca. Nitrosocosmicus arcticus Kfb
Ca. Nitrosocosmicus exaquare
Ca. Nitrosocosmicus franklandus
Ca. Nitrososphaera gargensis Ga9.2
Nitrososphaera viennensis EN76
Ca. Nitrososphaera evergladensis SR1
RRmetagenome bin19
UBA536
UBA348
13 1 40CM 38 12
13 1 40CM 4 38 7
13 1 40CM 3 38 6
Ca. Nitrosotalea sinensis Nd2
Ca. Nitrosotalea bavarica SbT1
Ca. Nitrosotalea devanaterra
Ca. Nitrosotalea okcheonensis CS
NPMR_NP_iota_1
YK1309_1N_NP_iota
Ca. Nitrosotenuis chungbukensis MY2
Ca. Nitrosotenuis uzonensis N4
Ca. Nitrosotenuis cloacae
Ca. Nitrosotenuis aquarius
UBA219
UBA459
UBA218
Archaeon CSP1
NPMR_NP_delta_1
NPMR_NP_delta_3
NPMR_NP_delta_2
Ca. Nitrosopelagicus brevis
md1 - D11 MAG
og1 SAG - G13
mg3 - D17
mg4 - T3L15
og2 SAG - F20
SCGC AB-629-I23
AAA007-O23
NPMR_NP_theta_4
NPMR_NP_theta_3
YK1312_12N_NP_theta
NPMR_NP_theta_2
NPMR_NP_theta_1
NPMR_NP_theta_5
Cenarchaeum symbiosum A
Ca. Nitrosoarchaeum koreensis MY1
Ca. Nitrosoarchaeum limnia SFB1
Ca. Nitrosoarchaeum limnia BG20
casp-thauma3
ma1 - T1 L11
ma7 - T3L14
ma2 - D1
ma8 - T3L19
Ca. Nitrosopumilus koreensis AR1
Ca. Nitrosopumilus sp SJ
Ca. Nitrosopumilus piranensis
Nitrosopumilus maritimus SCM1
SCGC AAA799-E16
SCGC AAA799-N04
SCGC RSA3
SCGC AAA799-D11
NORP47
NP142
Ca. Nitrosomarinus catalina
casp-thauma1
Baikal-G2
Ca. Nitrosopumilus salaria BD31
Ca. Nitrosopumilus sediminis
CG 4 9 14 0 2 um filter 34 16
Ca. Nitrosopumilus sp. NM25
Ca. Nitrosopumilus adriaticus
Ca. Nitrosopumilus strain DDS1
nit2
ureA
mco1
larA
fdh
pgi
putA/proDH
rocA
oat
ggt
kal
kat
argD
serC
ilvA
ilvE
aspC
gcvTPH
metH
metE
pepB
pepN
pepF
M10
AprE
Sto/Pro
zincin
Do
PP2A
APC
ZIP
SSS-Urea
DAACS
ArsB
SBT
CUT1
CUT2
HAAT
PhnT
PiT
PhoT
QAT
BCCT
tbp
rpoS54
polD2
uvrABC
hef
phr
udg4
udg5
mpg
alkA
ogg1
tag
endoV
POP4
uspA
pcm
MIP
mscL
mscS
proP
nhaP
Trk
ectC
ipct/dipps
cspC
cshA
LLM
nanM
A-type_ATPase
V-type_ATPase
flaK
flaI/TadC
cheAB
cheY
xerD/xerC
>=6
5
4
3
2
1
Number of proteins

Figure S3
