# Supplementary material for: Genomes of Thaumarchaeota from deep sea sediments reveal specific adaptations of three independently evolved lineages
Source: ISME J. 2021 Apr 1;15(9):2792–808. doi: 10.1038/s41396-021-00962-6 (PMC8397731; doi:10.1038/s41396-021-00962-6)
Supplement: Supplementary file 6 — Figure S5 [file 41396_2021_962_MOESM6_ESM.pdf]

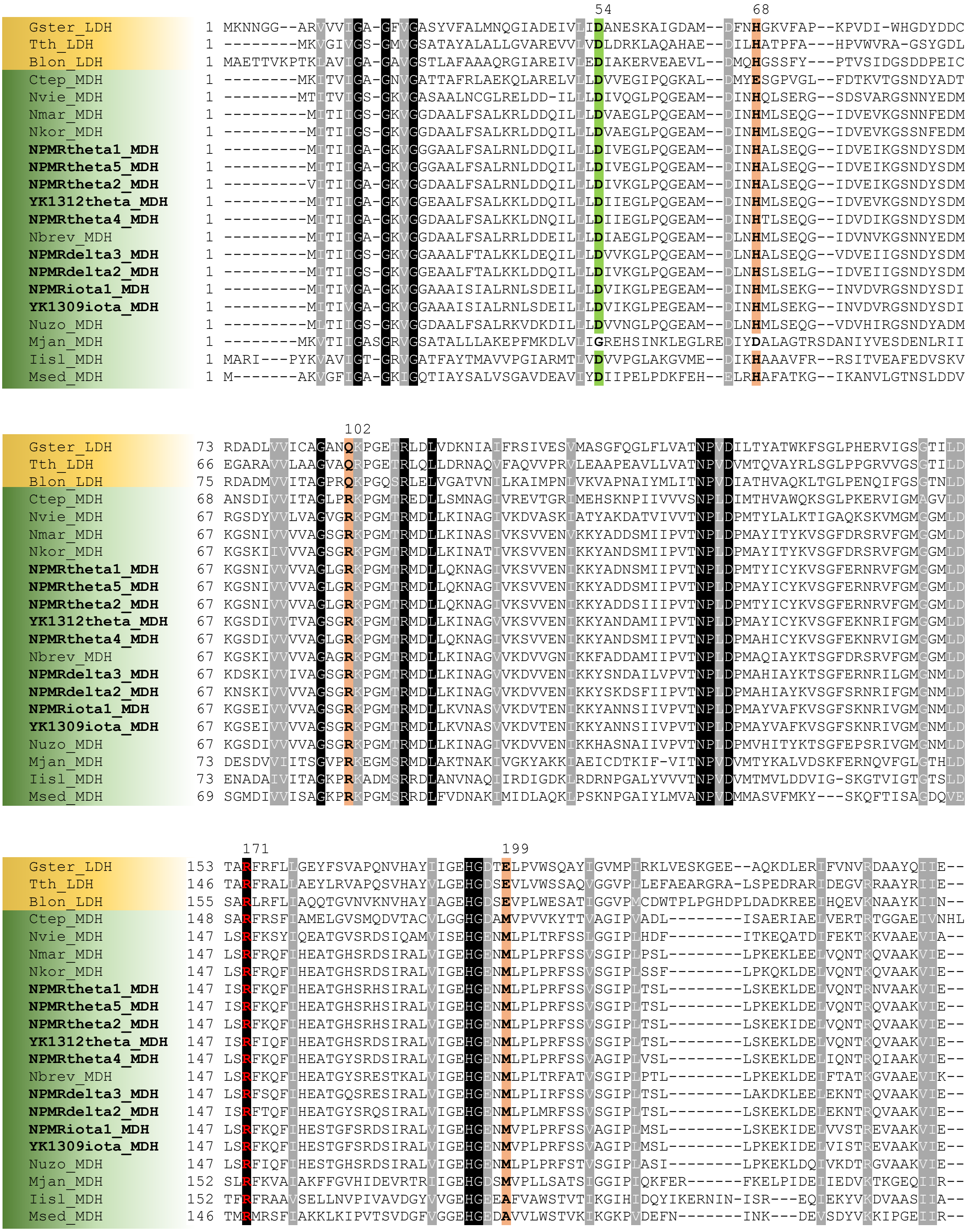
54 68
Gster_LDH 1 MKNNGG--ARVVVIGA-GFVGASYVFALMNQGIADEIVLIDANESKAIGDAM--DFNHGKVFAP--KPVDI-WHGDYDDC
Tth_LDH 1 --------MKVGIVGS-GMVGSATAYALALLGVAREVVLVDLDRKLAQAHAE--DILHATPFA---HPVWVRA-GSYGDL
Blon_LDH 1 MAETTVKPTKLAVIGA-GAVGSTLAFAAAQRGIAREIVLEDIAKERVEAEVL--DMQHGSSFY---PTVSIDGSDDPEIC
Ctep_MDH 1 --------MKITVIGA-GNVGATTAFRLAEKQLARELVLLDVVEGIPQGKAL--DMYESGPVGL--FDTKVTGSNDYADT
Nvie_MDH 1 --------MTITVIGS-GKVGASAALNCGLRELDD-ILLLDIVQGLPQGEAM--DINHQLSERG--SDSVARGSNNYEDM
Nmar_MDH 1 ---------MITIIGS-GKVGGDAALFSALKRLDDQILLLDVAEGLPQGEAM--DINHMLSEQG--IDVEVKGSNNFEDM
Nkor_MDH 1 ---------MITIIGS-GKVGGDAALFSALKRLDDQILLLDVAEGLPQGEAM--DINHMLSEQG--IDVEVKGSSNFEDM
NPMRtheta1_MDH 1 ---------MITIIGA-GKVGGGAALFSALRNLDDQILLLDIVEGLPQGEAM--DINHALSEQG--IDVEIKGSNDYSDM
NPMRtheta5_MDH 1 ---------MITIIGA-GKVGGGAALFSALRNLDDQILLLDIVEGLPQGEAM--DINHALSEQG--IDVEIKGSNDYSDM
NPMRtheta2_MDH 1 ---------VITIIGA-GKVGGDAALFSALRNLDDQILLLDIVKGLPQGEAM--DINHALSEQG--IDVEIKGSNDYSDM
YK1312theta_MDH 1 ---------MITIIGA-GKVGGEAALFSALKKLDDQILLLDIIEGLPQGEAM--DINHMLSEQG--IDVEVKGSNDYSDM
NPMRtheta4_MDH 1 ---------MITIIGA-GKVGGDAALFSALKKLDNQILLLDIIEGLPQGEAM--DINHTLSEQG--IDVDIKGSNDYSDM
Nbrev_MDH 1 ---------MITIIGA-GKVGGDAALFSALRRLDDEILLLDIAEGLPQGEAM--LNHMLSEQG--IDVNVKGSNNYEDM
NPMRdelta3_MDH 1 ---------MITIIGS-GKVGGAAALFTALKKLDEQILLLDVVKGLPQGEAM--DLNHALSEQG--VDVEIIGSNDYSDM
NPMRdelta2_MDH 1 ---------MITIIGS-GKVGGEAALFTALKKLDDQILLLDIVKGLPQGEAM--DLNHSLSELG--IDVEIIGSNDYSDM
NPMRiota1_MDH 1 ---------MITIVGA-GKVGGAAAISIALRNLSDEILLLDVIKGLPEGEAM--DINHMLSEKG--INVDVRGSNDYSDI
YK1309iota_MDH 1 ---------MITIVGA-GKVGGAAAISIALRNLSDEILLLDVIKGLPEGEAM--DINHMLSEKG--INVDVRGSNDYSDI
Nuzo_MDH 1 ---------MLTIIGS-GKVGGDAALFSALRKVDKDILLLDVVNGLPQGEAM--DLNHMLSEQG--VDVHIRGSNDYADM
Mjan_MDH 1 --------MKVTIIGASGRVGSATALLLAKEPFMKDLVLIGREHSINKLEGLREDIYDALAGTRSDANIYVESDENLRII
Iisl_MDH 1 MARI---PYKVAVIGT-GRVGATFAYTMAVVPGIARMTLVDVVPGLAKGVME--DIKHAAAVFR--RSITVEAFEDVSKV
Msed_MDH 1 M-------AKVGFIGA-GKIGQTIAYSALVSGAVDEAVIYDIIPELPDKFEH--ELRHAFATKG--IKANVLGTNSLDDV
102
Gster_LDH 73 RDADLVVICAGANQKPGETRLDLVDKNIAIFRSIVESVMASGFQGLFLVATNPVDILTYATWKFSGLPHERVIGSGTILD
Tth_LDH 66 EGARAVVLAAGVAQRPGETRLQLLDRNAQVFAQVVPRVLEAAPEAVLLVATNPVDVMTQVAYRLSGLPPGRVVGSGTILD
Blon_LDH 75 RDADMVVITAGPRQKPGQSRLELVGATVNILKAIMPNLVKVAPNAIYMLITNPVDIATHVAQKLTGLPENQIFGSGTNLD
Ctep_MDH 68 ANSDIVVITAGLPRKPGMTREDLLSMNAGIVREVTGRIMEHSKNPIIVVVSNPLDIMTHVAWQKSGLPKERVIGMAGVLD
Nvie_MDH 67 RGSDYVVLVAGVGRKPGMTRMDLLKINAGIVKDVASKIATYAKDATVIVVTNPLDPMTYLALKTIGAQKSKVMGMGGMLD
Nmar_MDH 67 KGSKIVVVVAGSGRKPGMTRMDLLKINASIVKSVVENVKKYADDSMIIPVTNPLDPMAYITYKVSGFDRSRVFGMGGMLD
Nkor_MDH 67 KGSKIIVVVAGSGRKPGMTRMDLLKINATIVKSVVENIKKYANDSMIIPVTNPLDPMAYITYKVSGFDRSRVFGMGGMLD
NPMRtheta1_MDH 67 KGSNIVVVVAGLGRKPGMTRMDLLQKNAGIVKSVVENIKKYADNSMIIPVTNPLDPMTYICYKVSGFERNRVFGMGGMLD
NPMRtheta5_MDH 67 KGSNIVVVVAGLGRKPGMTRMDLLQKNAGIVKSVVENIKKYADDSMIIPVTNPLDPMAYICYKVSGFERNRVFGMGGMLD
NPMRtheta2_MDH 67 KGSNIVVVVAGLGRKPGMTRMDLLQKNAGIVKSVVENIKKYADDSIIIPVTNPLDPMTYICYKVSGFERNRVFGMGGMLD
YK1312theta_MDH 67 KGSDIVVTVAGSGRKPGMTRMDLLKINAGVVKSVVENIKKYANDAMIIPVTNPLDPMAYICYKVSGFEKNRIFGMGGMLD
NPMRtheta4_MDH 67 KGSDIVVVVAGLGRKPGMTRMDLLQKNAGIVKSVVENIKKYANDSMIIPVTNPLDPMAHICYKVSGFEKNRVIGMGGMLD
Nbrev_MDH 67 KGSKIVVVVAGAGRKPGMTRMDLLKINAGVVKDVVGNIKKFADDAMIIPVTNPLDPMAQIAYKTSGFDRSRVFGMGGMLD
NPMRdelta3_MDH 67 KDSKIVVIVAGSGRKPGMTRMDLLKINAGVVKDVVENIKKYSNDAILVPVTNPLDPMAHIAYKTSGFERNRILGMGNMLD
NPMRdelta2_MDH 67 KNSKIVVVVAGSGRKPGMTRMDLLKINAGVVKDVVENIKKYSKDSFIIPVTNPLDPMAHIAYKVSGFSRNRIFGMGNMLD
NPMRiota1_MDH 67 KGSEIVVVVAGSGRKPGMTRMDLLKVNASVVKDVTENIKKYANNSIIVPVTNPLDPMAYVAFKVSGFSKNRIVGMGNMLD
YK1309iota_MDH 67 KGSEIVVVVAGSGRKPGMTRMDLLKVNASVVKDVTENIKKYANNSIIVPVTNPLDPMAYVAFKVSGFSKNRIVGMGNMLD
Nuzo_MDH 67 KGSDIVVVVAGSGRKPGMTRMDLLKINAGIVKDVVENIKKHASNAIIVPVTNPLDPMVHITYKTSGFEPSRIVGMGNMLD
Mjan_MDH 73 DESDVVIITSGVPRKEGMSRMDLAKTNAKIVGKYAKKIAEICDTKIF-VITNPVDVMTYKALVDSKFERNQVFGLGTHLD
Iisl_MDH 73 ENADAIVITAGKPRKADMSRRDLANVNAQIIRDIGDKLRDRNPGALYVVVTNPVDVMTMVLDDVIG-SKGTVIGTGTSLD
Msed_MDH 69 SGMDIVVISAGKPRKPGMSRRDLFVDNAKIMIDLAQKLPSKNPGAIYLMVANPVDMMASVFMKY---SKQFTISAGDQVE
171 199
Gster_LDH 153 TARFRFLLGEYFSVAPQNVHAYIIGEHGDTELPVWSQAYIGVMPIRKLVESKGEE--AQKDLERIFVNVRDAAYQIIE--
Tth_LDH 146 TARFRALLAEYLRVAPQSVHAYVLGEHGDSEVLVWSSAQVGGVPLLEFAEARGRA-LSPEDRARIDEGVRRAAYRIIE--
Blon_LDH 155 SARLRFLIAQQTGVNVKNVHAYIAGEHGDSEVPLWESATIGGVPMCDWTPLPGHDPLDADKREEIHQEVKNAAYKIIN--
Ctep_MDH 148 SARFRSFIAMELGVSMQDVTACVLGGHGDAMVPVVKYTTVAGIPVADL--------ISAERIAEVERTRTGGAEIVNHL
Nvie_MDH 147 LSRFKSYIQEATGVSRDSIQAMVISEHGENMLPLTRFSSLGGIPLHDF--------ITKEQATDIFEKTKKVAAEVIA--
Nmar_MDH 147 LSRFRQFIHEATGHSRDSIRALVIGEHGENMLPLPRFSSVSGIPLPSL--------LPKEKLEELVQNTKQVAAKVIE--
Nkor_MDH 147 LSRFRQFIHEATGHSRDSIRALVIGEHGENMLPLPRFSSVSGIPLSSF--------LPKQKLDELVQNTKQVAAKVIE--
NPMRtheta1_MDH 147 ISRFKQFIHEATGHSRHSIRALVIGEHGENMLPLPRFSSVSGIPLTSL--------LSKEKLDELVQNTRNVAAKVIE--
NPMRtheta5_MDH 147 ISRFKQFIHEATGHSRHSIRALVIGEHGENMLPLPRFSSVSGIPLTSL--------LPKEKLDELVQNTRQVAAKVIE--
NPMRtheta2_MDH 147 LSRFKQFIHEATGHSRHSIRALVIGEHGENMLPLPRFSSVSGIPLTSL--------LSKEKIDELVQNTRQVAAKVIE--
YK1312theta_MDH 147 ISRFIQFIHEATGHSRDSIRALVIGEHGENMLPLPRFSSVSGIPLTSL--------LSKEKLDELVQNTKQVAAKVIE--
NPMRtheta4_MDH 147 LSRFKQFIHEATGYSRDSIRALVIGEHGENMLPLPRFSSVAGIPLVSL--------LSKEKLDEIQNTRQIAAKVIE--
Nbrev_MDH 147 LSRFKQFIHEATGYSRESTKALVIGEHGENMLPLTRFATVSGIPLPTL--------LPKEKLDELFTATKGVAAEVIK--
NPMRdelta3_MDH 147 LSRFSQFIHEATGQSRESIRALVIGEHGENMLPLIRFSSVSGIPLTSL--------LAKDKLEELEKNTRQVAAKVIE--
NPMRdelta2_MDH 147 ISRFTQFIHEATGYSRQSIRALVIGEHGENMLPLMRFSSVSGIPLTSL--------LSKEKLDELEKNTRQVAAKVIE--
NPMRiota1_MDH 147 LSRFKQFIHESTGFSRDSIRALVIGEHGENMVPLPRFSSVAGIPLMSL--------LSKEKIDELVVSTREVAAKVIE--
YK1309iota_MDH 147 LSRFKQFIHESTGFSRDSIRALVIGEHGENMVPLPRFSSVAGIPLMSL--------LPKEKIDELVISTREVAAKVIE--
Nuzo_MDH 147 LSRFIQFIHESTGHSRDSIRALVIGEHGENMLPLPRFSTVSGIPLASI--------LPKEKLDQIVKDTRGVAAKVIE--
Mjan_MDH 152 SLRFKVAIAKFFGVHIDEVRTRIIGEHGDSMVPLLSATSIGGIPIQKFER------FKELPIDEIIEDVKTKGEQIIR--
Iisl_MDH 152 TFRFRAAVSELLNVPIVAVDGYVVGEHGEEAFVAWSTVTIKGIHIDQYIKERNIN-ISR---EQIEKYVKDVAASIIA--
Msed_MDH 146 TMRMRSFIAKKLKIPVTSVDGFVGGEHGEDAVVLWSTVKIKGKPVDEFN-------INK---DEVSDYVKKIPGEIIR--

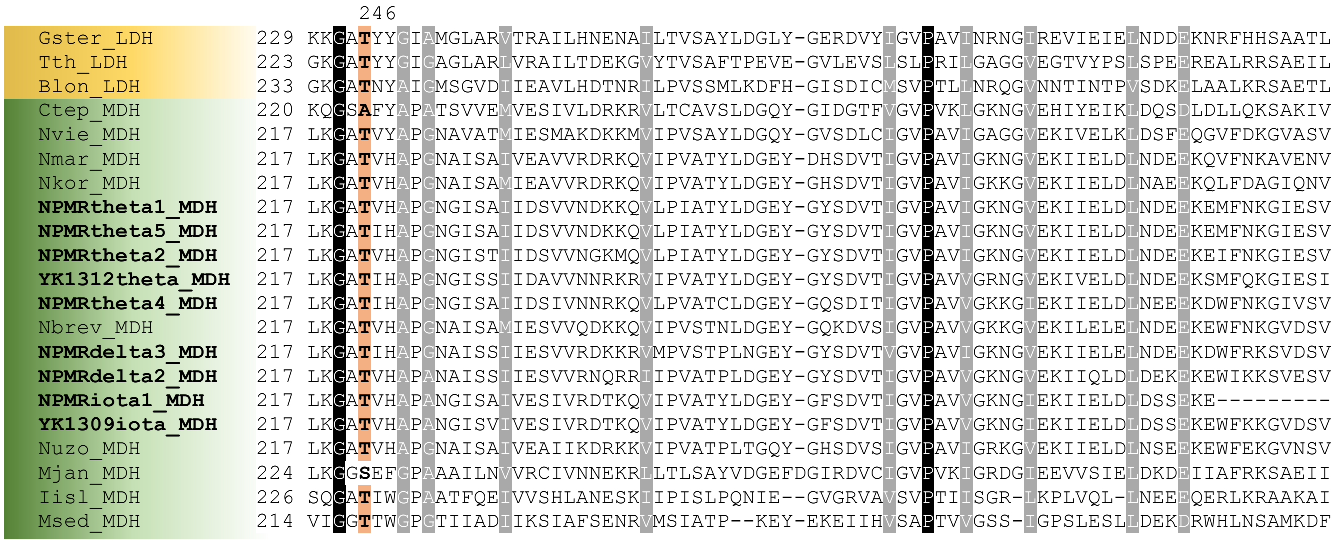

Fig. S5. Sequence alignment of lactate dehydrogenase (LDH) and malate dehydrogenase (MDH) homologs, shaded in yellow and green, respectively. Conserved residues are shaded in black and grey. The universally conserved in the LDH/MDH superfamily substrate binding Arg171 is indicated in red. Residues important for substrate discrimination and active site architecture are shaded in orange and discussed in the text. The residue determining the cofactor specificity is shaded in green. Residue numbering refers to the LDH numbering as in Roche *et al.* 2019.

Primary sequences from the following organisms were used to generate the alignment (Uniprot accession numbers in parentheses): Gster, *Geobacillus stearothermophilus* (P00344); Tth, *Thermus thermophilus* (Q5SJA1); Blon, *Bifidobacterium longum* (E8ME30); Ctep, *Chlorobaculum tepidum* (P80039); Nvie, *Nitrososphaera viennensis* (A0A060HG74); Nmar, *Nitrosopumilus maritimus* (A9A450); Nkor, *Nitrosarchaeum koreense* (F9CUM5); Nbrev, *Ca.* Nitrosopelagicus brevis (A0A0A7V4F4); Nuzo, *Ca.* Nitrosotenuis uzonensis (V6AR53); Mjan, *Methanocaldococcus jannaschii* (Q60176); Iisl, *Ignicoccus islandicus* (A0A0U3FQH7); Msed, *Metallosphaera sedula* (A4YDY0). Sequences from the marine sediment AOA MAGs reported in this study are in bold and their locus tags can be found in Table S3.
